# From a documented past of the Jersey breed in Africa to a profit index linked future

**DOI:** 10.3389/fgene.2022.881445

**Published:** 2022-10-28

**Authors:** Oluyinka Opoola, Felicien Shumbusho, David Hambrook, Sam Thomson, Harvey Dai, Mizeck G. G. Chagunda, Jude L. Capper, Dominic Moran, Raphael Mrode, Appolinaire Djikeng

**Affiliations:** ^1^ Centre for Tropical Livestock Genetics and Health (CTLGH), The Roslin Institute, University of Edinburgh, Edinburgh, United Kingdom; ^2^ Rwanda Agriculture and Animal Resources Development Board (RAB), Kigali, Rwanda; ^3^ Royal Jersey Agricultural and Horticultural Society (RJAHS), Trinity, Jersey; ^4^ Land O’Lakes Venture37®, Arden Hills, MN, United States; ^5^ Animal Breeding and Husbandry in the Tropics and Subtropics, University of Hohenheim, Stuttgart, Germany; ^6^ Livestock Sustainability Consultancy, Oxfordshire, United Kingdom; ^7^ Global Academy of Agriculture and Food Security, University of Edinburgh, Edinburgh, United Kingdom; ^8^ Scotlands’ Rural College, Roslin Institute Building, Edinburgh, United Kingdom; ^9^ International Livestock Research Institute (ILRI), Nairobi, Kenya

**Keywords:** Rwanda, dairy profit index, feed efficiency, milk yield, fertility, Jersey breed

## Abstract

The paper reports on the prevalence and performance of the Jersey cattle breed in Africa, highlighting its geographic distribution and describing the reported performance and other related characteristics from the early 1900s to the present day. The review examines the contribution of Jersey cattle in increasing the volume and efficiency of milk production across the continent. Data relating to the Jersey cattle breed has been reported in more than 30 African countries based on available material published between 1964 and 2020. A key encompassing parameter of any reference was a well-described consideration of the Jersey cattle breed (as pure or crossbred with other exotic and/or indigenous breeds) with reported performance within a variety of production systems and agro-ecologies in Africa. The main focus was on breed and performance parameters, breed types, percentage of different breed types in specific environments, reproduction method and fertility; survival and longevity; disease incidence; and production efficiency metrics such as: feed efficiency (milk unit per dry matter intake, DMI) and milk yield (MY) per unit of body weight (BW). The main performance descriptors identified were based on observations on resilience under both abiotic (heat, nutrition) and biotic (incidences of pests and diseases) stressors, milk production, BW, nutrition and utilisation of feed resources. From the literature consulted, we grouped key dairy cattle performance characteristics reported in each country under the following areas to aid comparisons; a. Milk production (Milk nutrient value, daily MY, lifetime MY and annual MY); b. Fertility traits and AFC; c. Survival and longevity, d. Production efficiency (Feed efficiency, milk per unit BW and milk per unit DMI and e. Disease incidences. Results of the review showed that the smaller stature and lower maintenance nutrient requirements of the Jersey breed means that it is better suited to tolerate the tropical production conditions in the African small-scale dairy farming sector. Detailed analyses on MY and survival showed that Jersey crosses with exotic and African indigenous breeds performed better than purebred cattle with strong evidence to support the suitability of the Jersey breed in crossbreeding with indigenous breeds for use in smallholder production systems.

## Introduction

The Jersey cattle breed originates from Jersey Island (a small British island found in the English Channel, close to the French coast), where Jersey cattle are still found today in purebred herds (Buchanan, 2002; Huson et al., 2020). It is the smallest of the common European dairy breeds and has been reported as a highly prized productive cow for centuries and as a distinct breed with a recorded history for nearly 200 years (JerseyCanada, 2019). Notwithstanding its origin on a small island, the Jersey breed has been exported to nearly all parts of the world for dairy development over the past century (Becker, 1973; JerseyCanada, 2019). Numerous benefits of the Jersey breed have been reported in the global dairy industry. The first reported introduction of the Jersey cattle to Africa dates back to the 1880s, nearly 140 years ago (Willis, 2012; Britannica, 2019). Over time, both formal and informal observations have been carried out relating to specific parameters/traits and the overall performance of the Jersey breed. Some of these observations supported genetic improvement programmes through crossbreeding of Jersey (exotic) animals with locally adapted or native breed cows and, more recently, have been used as the foundations for long-term genetic improvement programmes in Africa (Marshall et al., 2019). Other introductions of Jersey cattle to Africa have been opportunistic and not deliberately aligned with any national dairy improvement strategy (Dessie and Mwai, 2019). To contribute to this knowledge generation, we reviewed the distribution of Jersey cattle, evaluated key performance and resilience indicators, and discussed the findings within the context of the Jersey being suitable for low-input smallholder dairy production systems in Africa.

African livestock contribute 30–40 percent of the agricultural Gross Domestic Product (AgGDP; FAO, 2019) and are a vital source of nutrients. Globally, livestock products (e.g. milk, meat and eggs) contribute about 13% of the world’s calorie intake, yet, more importantly, serve as rich sources of protein and essential amino acids (FAO, 2009; FAO-GFFA, 2018). Considerable research has been undertaken to improve the nutrition of some of the world’s poorest people (Neumann et al., 2007; Randolph et al., 2007; Smith et al., 2012; Sibhatu et al., 2015). In Africa, livestock production must increase to meet the growing demands for milk, meat and eggs. Population growth and socio-economic development in Africa are driving important societal changes including increased disposable income, changes in nutritional and dietary needs and desires, and increased urbanisation that support the need for improved livestock production systems. Indeed, the FAO has estimated that global food supplies will have to increase by 60% in the next 30 years to support this demand (FAO, 2013a). As a result, livestock producers and food system stakeholders will have to make significant investments in key sectors of animal agriculture, including dairy.

One major challenge of livestock development in Africa and other low- and middle-income countries (LMICs) is to sustainably close productivity gaps which, in terms of milk production per cow (productive efficiency) is currently about 10-fold below the levels routinely achieved in Europe (FAOSTAT, 2019). Another major challenge is the potential negative environmental impacts of livestock and increased use of resources for agricultural production. According to the FAO (2013b), the livestock sector contributes 14.5% of global greenhouse gas (GHG) emissions, potentially exacerbating climate change and environmental variability. This is exacerbated by the relatively greater proportions of methane (CH_4_) and nitrous oxide (N_2_O) in the total GHG emissions from ruminant livestock, both gases being considerably more potent drivers of global warming than carbon dioxide (Thornton and Herrero, 2010). Inevitably and very importantly, an improved livestock sector therefore plays a crucial role in mitigating GHG emissions (Rojas-Downing et al., 2017). Africa as a continent relies on livestock, ecosystem goods for livelihood and has a less developed agricultural production system than in more developed countries (Herrero et al., 2013a).

For low-income countries of Africa and Asia, the largest part of GHG emissions originates from Agriculture, Forestry and Other Land Use; AFOLU (Herrero et al., 2013b; Pradhan et al., 2019) and the rest originates from urban activities, energy and industry and other sources (Osman-Elasha and de Velasco, 2020). For high-income countries, GHG emissions originate mainly from sources related to energy supply and industry (Osman-Elasha and de Velasco, 2020). These GHG emission intensities are driven by low animal productivity across large areas of arid lands, the use of poor quality feeds, feed scarcity, and animals with low productive potential that are often used for draft power and to manage household risk, as well as for production. A recent study by reported that mitigating environmental footprints in Africa should be in confluence with increasing livestock efficiency and productivity so that the proportion between GHG emissions per unit of product is reduced to similar outcomes available in other regions (e.g. Europe). As a strategy to reduce emissions and climate change impacts in the continent, Africa signed a formal consent and treaty under the Paris Agreement in 2017 to combat these issues (UNFCCC, 2020). The estimated emission at the time was approximately 4% compared to over 80% contributions from developed regions. However, with projections in population growth, urbanisation, financial growth and affluence, Africa’s emissions may rise by 30% in the coming decades which would be contrary to the findings envisaged under the Paris Agreement (Leon et al., 2021).

For the sustainability of implementation plans to reduce emissions, Africa proceeded to formulate polices to combat consequences of climate change without any delay and ahead of the 26th UN Climate Change Conference of the Parties (COP26) Summit in 2021 (Leon et al., 2021). The COP26 summit brought all parties, represented as countries, together to accelerate practical actions towards the goals of the Paris Agreement and the UN Framework Convention on Climate Change. The policies are being implemented and adhered to in Africa at country level. As an example; Kenya, South Africa, Ghana, Democratic of Congo, Angola and Gambia have developed nationally determined contributions to counteract climate change, ensure accountability and transparency underpinned by comprehensive and effective national and regional policy planning, capacity-building initiatives and proper governance structures.

### The African dairy sector

The dairy sector in Africa involves three forms of systems; extensive, semi-intensive and intensive, which are also classified according to the type and level of inputs: as low, medium and high, e.g. an extensive system = low inputs; intensive = high inputs, *etc.* Dairy breeds within these systems may be exotic or indigenous: exotic breeds are mainly Holstein-Friesian, Jersey and Ayrshire, with very few Guernsey, Brown-Swiss or Dairy Shorthorn cattle. Indigenous breeds mainly consist of African *bos taurus*, *bos indicus* (Zebu) and Sanga breeds, e.g. Indian breeds, Ankole, Tuli, N’Dama, Boran Watusi, Nguni and others, which vary in use depending on the dairy systems and geographical region. The productivity of indigenous breeds is very low, ranging from a minimum of 0.5 L to a maximum of 6–8 L per day, depending on disease prevalence, climatic conditions, availability of feed and water, lactation cycle and parity of cows (Brown, 1959; Ngono et al., 2018). By contrast, exotic breeds could perform at much higher levels, but often do not exhibit their full genetic potential in African systems due to abiotic and biotic stresses and less than optimal management conditions.

Over the past two decades in sub-Saharan Africa (SSA), various national dairy development plans have been supported by development partners, philanthropists and non-governmental organisations (NGOs). From these interventions, cross breeding of exotic with local genetics has been widely used to improve productivity. However, joint efforts have tried (with varying levels of success) to improve dairy productivity in Africa by establishing centralised dairy improvement programmes with support from development agencies and government-led efforts. For SSA in general, cow milk production is predominant, followed by goat milk, sheep milk and camel milk (Bingi and Tondel, 2015). Despite the encouraging progress in the East African region, the success of centralised dairy breeding programmes has been variable due to a lack of clear and relevant breeding objectives and strategies that are specific to production systems (Ojango et al., 2019). Centralised dairy breeding programmes have the potential to contribute to genetic improvement of exotic, indigenous or crossbred animals using open or closed nucleus breeding herds and have shown productivity levels comparable to those seen under research conditions. However, there has been limited consideration of research into farmers’ perceptions of the resulting cattle, the key traits and characteristics of different breeds, and the alignment of the breeding programmes with researchers’ interests. Uncoordinated efforts have also led to inconsistent decisions on breed choices, leading to a poor match between the chosen dairy breeds and herd management systems in terms of optimum production and resilience (Bhuiyan et al., 2017; Alilo, 2019).

Interventions to improve dairy production in Africa have recently been reviewed and redefined with more impetus through the development of national dairy platforms and national livestock masterplans for instance in Uganda (Balikowa, 2011); Kenya (Bingi and Tondel, 2015); Rwanda (Shapiro et al., 2017a; Shapiro et al., 2017b); Tanzania (Michael et al., 2018). Additional efforts have supported strategic guidance through policies and support for animal tracing and performance data recording for efficient and sustained genetic progress (DDA, 2021) and the development of multi-stakeholder value chains and commercialisation of dairy products (Michael et al., 2018; Ojango et al., 2019). East Africa is the leading milk-producing region in Africa, accounting for 68% of the continent’s milk output (ILRI, 2013). The dairy sector is one of the fastest growing agricultural sub-sectors in Eastern African countries, which has generated significant economic returns and employment opportunities along dairy value chains (Makoni et al., 2013). Kenya and Tanzania are among the biggest dairy producers in Africa, but other countries, including Rwanda (MINAGRI, 2019) and Uganda (FAOSTAT, 2019), are on a trajectory for increased dairy production to meet the growing and increasing demand (DDA, 2021). Although Ethiopia has the largest dairy cattle population in Africa, productivity remains low (Getabalew et al., 2019).

The challenges facing dairy producers in Africa are numerous, complex and vary depending on countries, regions and management systems (Njonge, 2017; Opoola et al., 2019). These challenges are exacerbated by somewhat outdated views on breeding policy based on Western notions of more extreme purebred dairy exotic breeds as being the most suitable for dairying across the continent, with a focus on peak daily MY rather than lifetime or annual MY and without reference to the limitations placed on cattle performance by often inadequate feed resources.

### Highlights of the Jersey breed in selected African countries

Jersey cattle were first imported into Africa *via* South Africa in the 1880s and have since expanded into other African countries. Although no records are available to support the exact date of the first Jersey importation into South Africa, it is generally accepted that the first Jerseys were imported by Mr. Adrian van der Byl of Roodebloem Estate, Woodstock, Cape, from Jersey Island, in the early 1880s (Willis, 2012). Jersey heifers exhibit significant calving ease while calving and low calf mortality compared to other breeds (Dhakal et al., 2013). There is information suggesting that Jerseys are disease-resistant, thermo-tolerant and well adapted to challenges of the tropical environment, including limited water, sub-optimum nutrition, pests’ infestation, vector-borne diseases, heat stress, and other issues. Additionally, Jersey cattle are known to adapt well to many types of climate, environment and management practices (Porter et al., 2016).

With reference to the tropical environment, it would therefore appear that the Jersey is a suitable breed to help reduce the impact of genotype-by-environment (or GxE) interactions exhibited by other exotic dairy breeds currently used for dairy production systems in Africa (Jersey Finance, 2020); genotype-by-environment being defined as when two different genotypes respond to environmental variation in different ways (Fikse et al., 2003). Finally, the Jersey breed would appear to give the fastest returns and profit by 5 years of age and overall performance in fertility, survival and management traits analysed for Jersey than other exotic dairy breeds (Garcia-Peniche, 2004).

The aim of this paper was firstly; to review the documented reports, absence or presence of the Jersey breed in countries in Africa, including its performance in comparison to other breeds or crosses. Secondly; to identify important parameters that can be used for decision-support including building a profit (suitability) index for African countries.

## Methods

Our review focused on Jersey cattle documentation between 1964 and 2020. We performed a meta-analysis of over 200 documents including journal articles, conference papers, reports and “grey” literatures published from 1964 to 2020. We combined the internet searches of key science databases (Pubmed^®^, Google Scholar^®^, Web of Knowledge^®^) with documents from national archives (e.g. Jersey Island, Rwanda, Zambia, Lesotho, Swaziland, E-Swatini and Somalia). The search strategy employed included the following search terms: “Jersey,” “Jersey performance in low- and middle-income countries (LMICs),” or “Jersey for low-input systems,” in conjunction with the name of any African country (e.g. “Jersey breed performance in Mozambique”). The search was narrowed down to only include references that reported on the distribution, occurrence, breed characteristics, performance (particularly with regards to dairy production) and the search terms as mentioned above for Jersey cattle in Central, Eastern, Northern, Southern and Western Africa. Information from grey literature and archives were made available from the Royal Jersey Agricultural & Horticultural Society (RJAHS), Rwanda Agriculture and Animal Resources Development Board (RAB), Land O’ Lakes Venture 37^®^ and personal communications and experiences from key livestock scientists and development experts. Additional printed documents in the forms of reports and old journals with relevant information on Jersey cattle (including their crosses with indigenous cattle breeds and the recorded performances) were also consulted from RJAHS, online articles, newspapers and manually curated by the authors. For comparison, other references with information on Jersey cattle within Asia and Latin America were also considered. Descriptive statistics were calculated with R programme (R core team, 2015) to determine traits such as MY, AFC, calving interval, reproductive methods (AI and natural service) and BW for Jersey cattle across different African countries.

## Results

### Jersey distribution in Africa

**FIGURE 1:**
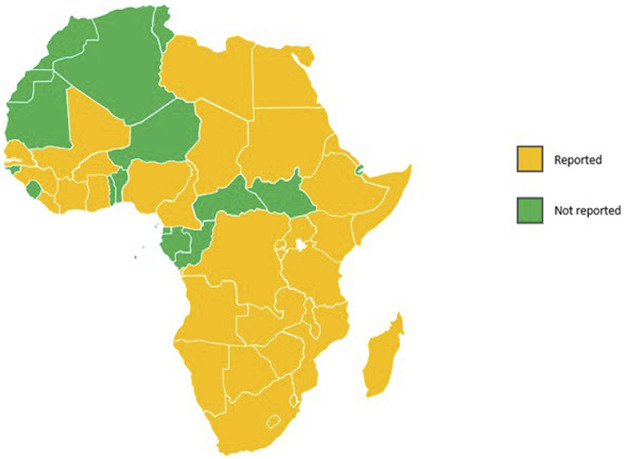
Map of Africa showing the presence of the Jersey breed.

We analysed the 200 documents generated from the searches. Based on our findings, the Jersey breed was reported (either currently or historically) in 34 African countries (Figure 1). The Jersey breed was reported either as purebred cattle, or crossbred with exotic or indigenous dairy breeds occurring at different genetic levels and contributing to the 10% to over 80% of other exotic and indigenous dairy cattle. It is however, highly probable that there are many more countries where Jersey cattle are likely to be present but just not reported as so in peer-reviewed literature. However, it would not be surprising if Jersey cattle, or at least Jersey genetics, existed in all African countries.

The countries reporting Jersey cattle present within their dairy populations and across many different management systems include: Angola, Botswana, Burkina Faso, Burundi, Cameroon, Chad, Cote d’Ivoire, Democratic Republic of the Congo, Egypt, Eritrea, E-Swatini, Ethiopia, Gambia, Guinea, Ghana, Lesotho, Kenya, Liberia, Libya, Madagascar, Malawi, Mozambique, Namibia, Nigeria, Rwanda, Senegal, Seychelles, Somalia, South Africa, Sudan, Tanzania, Uganda, Zambia and Zimbabwe.

### The proportion of Jersey cattle relative to other dairy breeds in Africa

In all countries reporting Jersey cattle, their proportion relative to other breeds is considerable. Recent data on Jersey breed proportions in other dairy herds in Africa is limited. Previous studies in Kenya (Kang’ethe et al., 2020), South Africa (Theron and Mostert, 2009), Ethiopia (Effa et al., 2013), Rwanda (Manzi et al., 2012), Sudan (Osman and Russel, 1974), Tanzania (Moyo and Mpofu, 1998), Cameroon (Djoko et al., 2003), Ivory Coast (Letenneur, 1978), Nigeria (Adebayo and Oseni, 2016), The Gambia (Diack et al., 2005), Malawi (Banda, 1996), Zimbabwe (Missanjo et al., 2012) and Egypt (Cunningham and Syrstad, 1987) have reported various descriptive statistics, genetic correlations and estimation of genetic parameters and variance components. Recently, genomic diversity and population structure of the Jersey breed amongst other breeds has been evaluated (Chagunda et al., 2018). South Africa and Kenya have extensively reported on the performance of Jersey breed with other breeds in diverse dairy production systems (Staal et al., 2001; Banga and Maiwashe, 2013; Kibiego et al., 2015). Comprehensive and detailed information on data types and evaluation types carried out in these countries is available in Opoola et al. (2021). This difference in cattle populations may reflect the relative intensification of dairy production in South Africa and Kenya compared to other African countries. In addition, genetic parameters such as; estimates for desirable and heritable traits, genetic correlation, genomic diversity and population structure have also been reported for Brown-Swiss and some Indigenous breeds in South Africa (de Ponte Bouwer et al., 2013; Makina, 2015). The proportion of Jersey cattle within national dairy populations relative to other breeds, across African countries other than Kenya or South Africa were not readily available at the time of carrying out this review, with no cited or reported information available in public domains. This lack of clarity on the extent of the Jersey population by country therefore leads to a call for improved data recording, monitoring and publication of Jersey cattle use in Africa’s dairy management systems. However, documented production and reproduction performance traits for other dairy breeds exist (Table 1), with cited and documented average (±standard deviation) performances of the Jersey breed amongst other dairy breeds in Africa.

**TABLE 1:** Evaluation types identified within this report for the Jersey breed performance with other exotic and indigenous breeds.

Traits	Breeds and admixture composition	Trait estimates	Data type	Evaluation type
Breed composition; Exotic breeds (JER, HOF, DSH, GUE, AYR); indigenous breeds (EAZ, Mpwapwa, Horro, Boran, Sahiwal, White Fulani, Red Sindhi, Ankole, *etc*.) and their crossbreds (exotic and Indigenous; JER crosses; HOF crosses, *etc*.)	*Bos taurus* to *Bos indicus* blood levels	12.5%; 25%; 50%; 75%; 85% and <85%	Production and fertility data	Preliminary analysis; REML; ANOVA
Reproduction method; Artificial insemination (AI), natural mating		<90% AI; <10% natural mating with exotic bull stud	Production and fertility data	Preliminary analysis
Body weight (kg)		350–420 kg	Production and fertility data	Descriptive statistics
305 Day Milk yield (L)	JER and JER crosses	1,683–5,000	Production and fertility data	Descriptive statistics; Genetic analyses
Calving interval (days)	JER and JER crosses	474	Production and fertility data	Descriptive statistics; Genetic analyses
Age at first calving (months)	JER and JER crosses	29–38	Production and fertility data	Descriptive statistics Genetic analyses
Feed efficiency	JER and JER crosses	-	Production, fertility and feed consumption data	Descriptive statistics; ANOVA
Character and temperament	JER and JER crosses	-	-	-
Disease	JER and JER crosses	-	Production and fertility data	Descriptive statistics
Adaptability	JER and JER crosses	-	Production and fertility data	Descriptive statistics; Genetic analyses
Lifespan/longevity	JER and JER crosses	-	-	Preliminary analysis

Breeds: JER, purebred Jersey; JER crosses, Crossbred Jersey; HOF, Holstein-Friesian; HOF crosses, Crossbred Holstein-Friesian; AYR, Ayrshire; BSW, Brown Swiss; DSH, Dairy Shorthorn; GUE, Guernsey; SAH, Sahiwal. The (-) implies no reported information available for the trait.

Table 2 shows favourable estimates demonstrating a Jersey and Jersey cross-breed advantage in pooled data analysed across the breeds for fertility traits such as; average number of completed lactations, age at first calving, first calving interval, average calving interval, number of inseminations per conception, feed efficiency and survival traits. Although pooled data for milk production, lifetime MY and CI were not always favourable in Jersey/Jersey-cross data compared to the Holstein-Friesian and Guernsey breeds, the data suggested that Jerseys and their crosses were more likely to attain shorter age at first calving, survive longer and complete more lactations over their lifetime than the other dairy breeds in most African dairy systems.

**TABLE 2:** Cited and documented average (±standard deviation) performances of Jersey breed amongst other dairy breeds in Africa.

	Dairy breeds							Reference(s)
Traits	JER	HOF	AYR	BSW	DSH	GUE	SAH	Meyn and Wilkins, (1974); Cunningham and Syrstad, (1987)
305-day MY (L)	4,666 ± 1,940	6,147 ± 2,131	3,565 ± 1,483	-	2,020	5,143 ± 252	893 ± 245	Meyn and Wilkins, (1974); Cunningham and Syrstad, (1987); Djoko et al., (2003)
1st MY (L)	4,113 ± 1,123	5,268 ± 1,879	1,842 ± 785	3,149		3,247 ± 779	-	Opoola et al., (2020); Chenyambuga and Mseleko, (2009)
Ave no. lactation completed	3.2	2.8	-	-	-		-	Theron and Mostert, (2009); Chenyambuga and Mseleko, (2009)
Calving to 1st heat interval (days)	80	69	-	-	-	-	-	Siyoum et al. (2016)
Gestation length (days)	283	282 ± 0.7	-	-	-	-	-	Siyoum et al. (2016)
Days open	123	143 ± 33		-	-	-	-	Mulangila, (1997); Siyoum et al., (2016); Asimwe and Kifaro, (2007); Chenyambuga and Mseleko, (2009)
AFC (days)	935 ± 28	1,029 ± 169	1,050 ± 130	-	1,086 ± 189	1,044 ± 134	1,169 ± 15	Njubi et al., (1992); Mulangila, (1997); Wakhungu et al., (2006); Opoola et al., (2020)
1st CI (days)	410 ± 21	466 ± 3	404			393	-	Mostert et al., (2010); Opoola et al., (2020)
CI (days)	401 ± 58	468 ± 18	418 ± 11	-	436–452	397 ± 10	493 ± 5	Kanuya and Greve, (2000); Mostert et al., (2010); Nandolo, (2015); Opoola et al., (2020)
Survival per lactation (%)	34	23	29	-	-	-	-	Phillips, (2014); Muller and de Waal, (2016)
Survival per year (%)	-	-	-	-	-	-	922	Wakhungu et al. (2006)
N^o^. of insemination per conception	1.94	1.96	2.17	-	-	-	-	Siyoum et al. (2016)
Feed efficiency (grams/L)	272	258	-	-	-	-	-	Phillips, (2014)
*Longevity (days)	3,722 ± 270	3,970 ± 237	-	-	-	-	-	Effa et al. (2013)

Breeds: JER, Jersey; HOF, Holstein-Friesian; AYR, Ayrshire; BSW, Brown Swiss; DSH, Dairy Shorthorn; GUE, Guernsey; SAH, Sahiwal; CI, Calving interval; AFC, Age at fist calving; MY, Milk yield; *Least square means (days), The (-) implies no reported information available for the traits.

### Phenotypic characteristics of Jersey dairy cattle

Based on the reviewed materials, and compared to other dairy breeds, the Jersey breed is reported to be hardy, resilient and adapted to a wide range of climatic and geographical conditions (Hilton and Briggs, 1980; Berry and Buckley, 2016) and diverse production systems (Effa et al., 2013; Huson et al., 2020). Morphologically, the Jersey breed appears in varied colours of dark brown to light brown, including strains that show white patches (Buchanan, 2002). The patches of white hair and lighter skin pigment (known as “broken coloured”) make these strains less well adapted for hotter climatic conditions due to greater susceptibility to sun exposure. All Jersey cattle have a characteristic black muzzle, surrounded by a mealy coloured band of hair and hard black hooves. These hard black hooves assist in minimising locomotion issues due to low housing spaces, poorly managed surfaces, with heavy rains causing soil erosion and sloping into where these cattle are kept. The Jersey cattle is habitually docile and inquisitive by nature, often dominating the social order and most always coexist with other larger dairy breeds (Phillips, 2014). This allow them to obtain a greater share of feed among other herds as well as better manageability and cooperation from the milking parlour (Jersey Finance, 2020). Although this is not necessarily an advantage in African systems *per se*, it means that the Jersey cattle may out-compete other breeds within the herd when resources are relatively scarce, as may occur in smallholder systems.

Jerseys are the smallest of the common exotic dairy breeds, weighing between 380 and 450 kg (Oklahoma State University Board of Regents, 2008) though more modern strains developed in the western hemisphere are larger, weighing up to 550 kg (Porter et al., 2016). The relatively lighter weight of Jersey cattle compared to many other breeds (Dhakal et al., 2013) is again an advantage in African systems where feed resources are scarce in most smallholder systems. A smaller animal needs less feed to maintain herself and is therefore more able to produce milk under conditions where feed resources may be limited, then her heavier counterparts (Vance et al., 2013). This also has environmental benefits as, per kg of milk produced, Jersey cattle have lower GHG emissions and requires fewer total resources (Capper and Cady, 2012).

In higher-income countries (Western Europe, North America and Australia), the dairy sector has made considerable progress in adopting genetics that confer advantages in body size, adaptability, resilience, productivity and quality of dairy products from breeds such as Jerseys because they are potentially more efficient than Holstein-Friesian cattle. Oldenbroek (1986) showed that the Jersey breed appeared to have a higher efficiency than expected; possibly due to the higher yield and feed intake per unit of BW compared to other breeds. Furthermore, Kasbergen (2013) indicated that compared with the Holstein, Jersey cows were more economically efficient, generating more income per kg of milk, due to the higher milk components (average solids non-fat% of 9.42% *versus* 8.78%), higher pregnancy rate, feed efficiency and increased income over feed cost (∼30%). The Jersey breed is able to convert low feed energy to an adequate milk volume and quality (Capper and Cady, 2012), which is especially important for smallholder farms that practice low-input dairy systems by default (Gollin, 2014; Abin et al., 2018). Furthermore, Jersey cattle show increased resilience to tick and vector-borne diseases aiding smallholder farms to reduce veterinary and other maintenance costs, and serve as a triple-purpose breed (dairy, meat and/or draught purposes) (Porter et al., 2016). The Jersey breed adapts well to the hot and dry environment with less of a compromise on milk performance and productivity, compared to other dairy breeds (Buchanan, 2002). The breed is also known to be cost-effective to manage and adapts well to a low-input system when compared to other exotic dairy breeds (Abin et al., 2018). Depending on the management system practiced, milk yield per unit of production input can be very cost-effective, providing an excellent source of nutrients for human consumption in addition to a potential source of income and revenue to meet smallholder farmers’ financial commitments (Herrero et al., 2014).

### Milk nutrient content, daily milk yield, annual milk yield and lifetime milk yield

The lifetime productivity of Jersey cattle will vary considerable depending on genetic merit, production system feed availability and quality, health and overall performance in different global regions. Although Jersey cows may produce less total milk on a daily basis than (Buchanan, 2002), for example, Holstein-Friesian cattle in European or North American systems, the increased milk solids content and resilience of the breed has significant impacts at the lifetime level, particularly in tropical or sub-tropical systems (Stelwagen, 2011; Nandolo, 2015). Krishanender et al. (2014) reported that lifetime productivity (whether measured as daily MY, annual MY or lifetime MY) was higher in pure and crossbred Jerseys than in other exotic or indigenous breeds in sub-temperate systems. Furthermore, Jersey cows have been reported to demonstrate significantly better lifetime daily yield (Boothby et al., 2020), age at first calving and survival rates (Buckley et al., 2014) compared to Holstein-Friesians in UK production systems. In dairy cows, certain terminologies often used interchangeably can be a bit confusing and ambiguous. Therefore, owing to the ambiguity, we define the following terms; longevity, herd life and productive life. Hu et al. (2021) defines longevity in dairy cows as the time from a cow’s first calving to when she exits the herd or does not have sufficient productivity. Herd life refers to the days from birth of a calf, produces her first calf; and to her culling or death (Hu et al., 2021; Zhang et al., 2021) and productive life refers to the days from the cow’s first calving to culling or death (Raguz et al., 2011). The proportion of days in milk over the total lifetime and the herd life of Jersey cattle were also increased compared to Holstein-Friesian, Brown-Swiss and Guernsey breeds (*p* < 0.01) in the study published by Garcia-Peniche (2004) for seven regions in the United States. With regards to Jersey crossbred cattle, Effa et al. (2013) reported better in the lifetime yield of F_1_ offspring of Jersey x Boran cows (13,546.50 ± 812.3 L) compared to F_1_ Holstein-Friesian × Boran cows (12,816.7 ± 817.0 L). The estimates for productive life, herd life, and AFC were also reported as more favourable for F_1_ Jersey × Boran crossbreds than in the F_1_ Holstein-Friesian × Boran crossbreds (Effa et al., 2013). Hunde et al. (2015) also observed a favourable mean AFC of 29.9 months (±0.17) in pure Jersey cattle compared to estimates of 40.9 months (±0.33) from Yalew et al. (2011) in pure Holstein-Friesian cattle managed in the Central Highlands of Ethiopia. However, after the F_1_ offspring, it is difficult to ascertain the genetic capacity and potential for productivity and fertility of subsequent generations (Alilo, 2018), as the Jersey genetics may be diluted out or affected by other breeds within the population.

Milk yields from Jersey cattle are in excess of 13 times their BW per lactation (David Clarke Livestock, 2021), a remarkable feat of efficiency given the increased milk fat and protein concentrations compared to other dairy breeds. For example, Bland et al. (2015) and Carroll et al. (2006) noted that Jersey milk contained 18% more protein, 25% more fat and 20% more calcium than milk produced from other dairy breeds; Holstein-Friesian and Brown-Swiss. This increase in milk solids content contributed to the greater cheese yield per kg of Jersey milk (compared to Holstein-Friesian milk) cited by Capper and Cady (2012) and therefore to improved production efficiency and reduced environmental impacts in North American production systems. This is of obvious importance from a food security and sustainability perspective within LMIC, as improving the nutritional status of some of the world’s poorest people leads to myriad health, development and social benefits.

Resistance to climate extremes is a key element of suitability for African production systems, with the most suitable cattle able to maintain productivity despite variation in temperature or humidity (Ekine-Dzivenu et al., 2020). A report by Phillips (2014) comparing heat stress responses in Jerseys and Holstein-Friesian dairy cows raised near the Mooi river of South Africa, showed that during the warmer months, Jersey cows exhibited a 5.35 L/cow/month reduction in total milk production compared to 5.76 L/cow/month in Holstein-Friesians, despite the higher genetic merit of the Holstein-Friesian cows. Moreover, the MY of Jersey and Holstein-Friesian cows on their third-and-over-lactation was 85% and 78%, respectively showing a remarkable yield persistence and improvement over time based on 305-day lactation (Phillips, 2014).

### Fertility traits and impact on age at first calving

From a lifecycle and efficiency point of view, the Jersey often has an advantage over larger breeds in terms of spending a greater proportion of her total life in lactation (Buchanan, 2002; Stelwagen, 2011). This is facilitated by an early age at puberty, better detection of oestrus behaviour, an early AFC and better calving interval, with a dry period that is suited to the herd and system (Parkinson et al., 2019). Traditionally, a 12-month calving interval has been considered to be ideal in many intensive dairy systems (Zeddies, 1982; Strandberg and Oltenacu, 1989), yet in dairy systems where feed or forage is limited, there may occasionally be some benefits to extending lactation if this results in a successful conception and pregnancy (Ratnayake et al., 1998). The bulk of the literature surveyed reported that purebred and crossbred Jersey cows reach puberty at an earlier age (Berry and Buckley, 2016) than other large sized exotic breeds, which may be a function of their smaller body size and therefore relatively higher body fat at a given age compared to larger-framed cattle. However, reproductive performance after puberty was also cited by Berry and Buckley (2016) as being better in Jersey cattle, with higher pregnancy rates, an earlier AFC and a reduced calving interval compared to other exotic or indigenous breeds. Conception rates and the number of inseminations per conception were also cited as improved in Jersey cattle, compared to other dairy breeds. Kasbergen (2013) reported that Jersey cows exhibited higher overall conception rate (CR) of 32% vs 29% CR for Holstein cows raised in the hot and dry climate of California, USA.

Dhakal et al. (2013) noted an improved ease for pure Jersey (JJ) and Holstein (HH) sires and dams mating to produce Jersey × Holstein (JH) and Holstein x Jersey (HJ) crosses, and other Jersey crosses (>50% JJ) in comparison with pure Holsteins and other Holstein crosses (>50% HH), in a study based on a pasture-based system in the USA. Pure Jerseys required calving assistance in only 7.5% of births from primiparous cows and 3.4% of births from multiparous cows, with Jersey crosses (>50%) requiring assistance in 8.3% of births from primiparous cows and 5.6% of births from multiparous cows. In comparison, calving assistance were more common in pure Holsteins (21.6% of births from primiparous cattle and 7.2% from multiparous), and in Holstein crosses (>50% HH) with 12.9% of primiparous births and 7.9% of multiparous births requiring assistance respectively. Crossing Jerseys directly with Holsteins also had a significant effect with assistance required in 8.8% (HJ) and 8.6% (JH) of births from primiparous cattle and 3.8% (HJ) and 4.8% (JH) of births in multiparous cattle. Calf mortality was also significantly lower in pure Jerseys (12.5% in primiparous cows and 5.6% in multiparous) compared with pure Holsteins (15.7% and 12.9% respectively).

The fertility attributes of the Jersey breed increases profitability of annual and lifetime milk production, longevity and number of subsequent calvings, as well as decreasing the time and impact on-farm resources (U.S. Jersey, 2014). Garcia-Peniche (2004) analysed fertility traits in Jersey cattle compared with other breeds in herds across multiple geographic and climatic regions of the USA and reported that in herds with a single breed of cattle, AFC in Jerseys averaged 778 (±3.1^1^) days, compared with 830 (±4.4) days for Brown Swiss and 803 (±3.0) days for Holsteins. In addition, the mean first calving interval in Jersey herds, measured in seven geographic regions, ranged from 390 (±5.1) days to 426 (±5.6) days, in comparison with a range across the same regions for Holstein herds of 409 (±3.4) days to 461 (±4.9) days. Evaluations of the performance of the Jersey breed in Africa by Opoola et al. (2020) also reported lower mean AFC in Jerseys compared with Holsteins in Kenya (909 days ± 31.44 for Jerseys vs. 972 days ± 3.93 for Holsteins) and South Africa (861 days ± 1.21 for Jerseys vs. 873 days ± 1.02 for Holsteins). In the same analysis, Jerseys also exhibited shorter mean calving intervals compared with Holsteins in both Kenya (457 days ± 28.77 for Jerseys, vs 475 days ± 6.12 for Holsteins) and South Africa (405 days ± 0.88 for Jerseys vs. 429 days ± 0.85 for Holsteins). Mostert et al. (2010) also showed decreases in the annual calving interval in Jersey cows (0.50 days/year) compared to increases in Holstein-Friesians (1.25 days/year), Ayrshire (0.71 days/year) and Guernseys (0.57 days/year). These would be expected to improve overall productivity and are thought to have been due to the inclusion of calving intervals and AFC standards in the selection of bull dams implemented by the Jersey Society since the early nineties in South Africa’s dairy breeding programme.

### Survival and longevity

The literature surveyed within this study showed that, compared to other breeds, Jersey cattle had improved survival-related traits in terms of longevity, herd life, the number of completed lactations and total days in milk (Wakhungu et al., 2006; Effa et al., 2013; Muller and de Waal, 2016). The longevity of dairy cattle attracts a great deal of debate worldwide, as there is no “ideal” number of lactations for a cow to complete within her lifetime (De Vries, 2020; Hu et al., 2021). The low number of lactations (1–3) completed by many cows in intensive systems attracts criticism, yet some researchers claim that keeping a cow for extended periods of time reduces the opportunity to make genetic gains (Capper and Cady, 2012; Parkinson et al., 2019). The decision of when to cull a cow is often based on economic factors (Lehenbauer and Oltjen, 1998). Therefore a breed like the Jersey, which is able to maintain productivity, longer life (more lactations), less need for replacement and a calf born every lactation that can be sold, can increase the total number of cows for smallholders. Less replacement costs for Jerseys compared to other breeds could be of economic and environmental value, as well as mitigating consumer concerns about cows being culled at relatively young ages. This is particularly important in smallholder systems in Africa as these cows are often the main source of income, status and high-quality protein (Ojango et al., 2019), therefore there are obvious economic, nutritional and social benefits to increased longevity. Muller and de Waal (2016) showed improved longevity and survival of first lactation cows to the fifth lactation at 34% for Jersey cows compared to 23% for Holstein cows bred in the Western Cape of South Africa. The effect of breed on longevity is not confined to African systems: research from the USA by Garcia-Peniche (2004) compared multiple longevity traits in herds of different breeds across geographic regions and reported increased average days of completed lactation in purebred Jersey herds with 633 (standard deviation SD; 291) days vs pure Brown-Swiss with 554 (SD 280.2) days and pure Holstein herds with 592 (SD 280) days. Jerseys also averaged increased survival rates in the herd from birth up to 5 years of age; 45% (SD 0.5) in pure Jersey herds vs. 38% (SD 0.49) in Holstein herds and 42% (SD 0.49) in Brown-Swiss herds.

Jersey crossbreds have also been demonstrated to perform favourably for longevity traits in tropical countries (Gebregziabher and Mulugeta, 2006; Effa et al., 2013; Hunde et al., 2015). In the tropical highlands of Ethiopia, estimates for longevity traits for F_1_ Jersey × Boran crosses showed significantly longer mean total life (4270 days ± 135), herd life (3108 days ± 147) and productive life (2387 days ± 126) when compared with F_1_ Friesian × Boran crosses (Effa et al., 2013). F_1_ Friesian × Boran crosses had mean total life of 4200 days (±135), mean herd life of 2877 days (±148), and mean productive life of 2145 days (±127). The F_1_ Jersey × Boran crosses also showed higher mean lifetime MY in litres (13547 ± 812, compared to 12817 ± 817 for F_1_ Friesian × Boran), though mean total MY in terms of litres per day of total life was broadly comparable at 3.04 L ± 0.2^2^ in F_1_ Jersey x Boran crosses vs 3.00 L ± 0.2^3^ in F_1_ Friesian × Boran crosses (Effa et al., 2013).

### Feed efficiency, milk per unit of bodyweight and milk per unit dry matter intake

Jerseys are efficient at converting feed into milk, which means that Jersey cows can produce a greater volume of milk per kg of DMI (Effa et al., 2013). This is a major advantage in terms of overall dairy sustainability, as feed efficiency has been cited as one of the key determinants of GHG emissions and resource use (Thoma et al., 2013), as well as farm profitability (Kasbergen, 2013). Carroll et al. (2006) reported that Jersey cows produced more fat corrected milk (FCM) and solids corrected milk (SCM) per kg of DMI than the Holstein and Brown-Swiss breeds. This was due to the greater efficiency of milk fat production per unit of DMI within Jersey cattle. In addition, Sneddon et al. (2011) reported that feed conversion efficiency (FCE) estimates, measured as grams of milk solids (milk fat plus milk protein) per kilogram of DMI were also higher in Jersey (112 g MS/kg DMI) than Holstein-Friesian cows (97 g MS/kg DMI). Sneddon et al. (2011) further showed that Jersey cows have significantly higher DMI per kilogram of BW compared to Holstein-Friesian and F_1_ of Holstein-Friesian × Jersey cows (3.81, 3.23 and 3.64 g DMI/kg BW, respectively); a result supported by Beecher et al. (2013). The small-framed Jersey cow has a lesser maintenance requirement than her large-framed herd mates. This favours her increased feed intake per unit of BW thus linking her ability to partition a greater proportion of feed nutrients into milk production.

This is referred to as the “dilution of maintenance” effect, whereby, as MY increases, the maintenance nutrient requirement is spread over the greater volume of milk, and therefore the nutrient use per kg of milk is reduced. This has significant environmental consequences, as discussed later in this report.

The greater milk fat yield of Jersey cows also has been linked with improved heterosis for milk fat yield genes in Jersey crossbreds, compared with other dairy breeds. Improved heterosis for fat yield percentage has been reported for Jersey × Boran crossbreds (5.10 ± 0.15%), by contrast to purebred Holstein-Friesian (4.77 ± 0.03%) and Boran cattle (5.01 ± 0.03%) under Ethiopian conditions (Hunde, 2019). This is of obvious advantage in terms of milk nutritional composition in its role in providing high-quality nutrition to smallholders and their families, but also in terms of commanding a greater price for milk sold for processing or consumption off-farm.

### Environmental impacts and sustainability of the Jersey breed

Jersey cattle exhibit a number of positive attributes in terms of productivity and efficiency, yet for a truly sustainable future, dairy producers must ensure that they have an economically viable, environmentally responsible and socially acceptable system in place. Although there is no “one size fits all” dairy system or collection of management practices that will results in sustainability for all farmers, the better an individual cow or herd can perform, the more sustainable it is likely to be. In this context, sustainability means using fewer resources (feed, land, fertilisers, fossil fuels) and having a lower carbon footprint (kg of GHG) per kg of solids-corrected milk. This should also result in a relatively lower cost of production, which is crucial for current and future economic viability, particularly in smallholder systems. Given that the concept of sustainability is a crucial dimension for all food systems, any production system that sets baseline and demonstrate improved sustainability is also likely to gain greater social acceptability. This is an obvious challenge in LMIC, where smallholders often lack access to the technological resources or infrastructure to assess the sustainability of their operation. Facilitating ways to measure and benchmark sustainability metrics on smallholder operations is therefore an important knowledge gap, which warrants significant investment.

The sustainability of dairy systems has been investigated by multiple authors with regards to genetics, nutrition, management and farming system, yet the data relating to sustainability of specific cattle breeds is lacking in the literature. The one exception is a paper by Capper and Cady (2012) which compared the environmental impacts of Jersey vs Holstein cattle under typical U.S. management systems. The study, a modelling exercise using publicly available data, quantitated the resource use and GHG emissions associated with producing the milk required to yield 500,000 t of cheese. Although Jersey cows had a lower daily MY than Holsteins (20.9 vs 29.1 kg), they were more efficient and had increased milk solids content for cheese yield, lower mature body weight and calving interval (8.0 kg milk/kg cheese; 454 kg BW; 13.7 months) than Holstein cows (9.9 kg milk/kg cheese; 680 kg BW; 14.1 months). In addition, Jersey cows exhibiting favourable age at first calving (25.3 vs. 26.1 months) coupled with improved longevity (3.00 vs. 2.54 lactations) meant that the Jersey cows had a greater production efficiency than their Holstein counterparts. Consequently, per kg of cheese yield, feed use was reduced by 19.8%, land use by 18.9%, water use by 31.6%, and the GHG emissions were 20.5% lower when milk from Jersey cattle was used rather than Holsteins. Although it was not quantified within the paper, the reductions in resource use per kg of cheese would also be expected to improve economic viability of Jersey compared to Holstein systems. It could be argued that the difference between Jersey and Holsteins might be less pronounced in a U.S. intensive system than in some of the far more extensive African conditions described within this review, therefore differences in the impacts described by Capper and Cady (2012) might be greater under tropical or sub-tropical conditions.

Various findings underline the suitability of Jersey cattle as a means to improve dairy sustainability through adaptation to the diverse production systems found across the globe. At present, smallholder systems are significantly disadvantaged when GHG emissions are used as the sole metric of assessing sustainability, as global analyses have reported that regions containing a high proportion of smallholder farming systems have greater carbon footprints per kg or ton of milk, meat or eggs (FAO, 2010; MacLeod et al., 2013; Opio et al., 2013). The current global standard for assessing greenhouse gas emissions is prescribed by the Intergovernmental Panel on Climate Change (IPCC, 2019) using three different types of calculations (Tiers I, II and III) to assess GHG emissions, depending on data availability. Tier I require the most basic data (total livestock numbers multiplied by a default emissions factor per head) and is used in many LMIC because it’s easy to apply. However, the default values used are based on intensive systems within developed regions, which cannot necessarily be applied to different systems or breeds (Leitner et al., 2021). Tier II is intermediate and Tier III is the most demanding in terms of complexity and data requirements. Both tiers are often referred to as higher tier methods utilised in most developed countries and are generally considered to be more accurate as adequate data are available to develop, evaluate and apply higher tier methods. More appropriate and accurate methane emissions factors must be calculated to be used on farms in LMIC, considering the efficiency and productivity benefits of Jersey cattle, in order to accurately assess the implications for GHG emissions for smallholders. Additional information on the performance and contribution of Jersey breed to dairy development across Africa is available in Opoola et al. (2021).

## Additional dimensions for harnessing the Jersey cattle in Africa

One important objective for conducting this review was to explore the opportunity for the development of a simple decision support tool (the Dairy Profit Index) and building on some key benefits of Jersey cattle as a critical contribution to profitable smallholder dairy systems in Africa. This review provides an assessment (albeit with limited, dated and sometimes less than reliable information) on the impact of the Jersey breed based on available references up to 2020 and recorded performances up to 2018. Our assessment could be considered biased as it was viewed in the context of adopting exotic and indigenous cattle breeds for previous and future dairy development strategies in Africa. Although the Jersey breed is present and actively used in many African countries, there is still a paucity of data available. For instance, Namibia has a strong livestock development plan and an emerging dairy sector; however, data on production and reproduction performance remains very limited. Similarly, Mozambique has a growing dairy sector with various crosses between the Jersey breed and indigenous breeds but the data is not yet available from purposefully designed studies to assess and support genetic improvement.

With the ever-increasing cost of feed and inputs, dairy farmers in climate-challenged regions of the world are beginning to think differently and explore opportunities to change cattle size, management systems, to improve financial status. Similarly, these trends are fast growing in Africa with smallholder farmers moving towards rearing medium-sized breed (e.g. Jersey) to drive milk output while maintaining cattle fertility and longevity (Okeyo, 2016; Okeyo, 2021). For instance, despite the abundance of other larger dairy breeds prevalent in Africa, the dairy sector still cannot meet the demand for dairy and dairy products (FAO, 2013a; FAO-GFFA, 2018). It is hypothesised that greater adoption of Jersey cattle in pure or crossbred form for dairying could help address issues relating to land size for dairying, land ownership, feed availability, community development and youth empowerment. In addition, it is proposed that an index mechanism or bio-economic model that factors profitability and sustainability of milk output that suits farmers’ current resources in Africa could support in aiding such a transition. The dairy sector in Africa is rapidly emerging and even re-emerging in various forms in many countries on the continent (Staal et al., 2001; Bingi and Tondel, 2015), yet the two primary commercial breeds (Holstein-Friesian and Jersey) are currently not farmed in purebred form (Gebrehiwot et al., 2020). The Jersey crossbreds have shown to be better adapted with a longer productive life than the Holstein-Friesian crossbreds (Okeyo, 2021). Therefore, it is important to explore the relevance of the characteristics of Jersey breed genetics for future dairy improvement strategies to ascertain what works best in terms of profit and revenue for the farmers, given the challenges of diverse production systems and climatic conditions.

Most dairy and beef markets have indexes that are mainly used to drive a farmer’s profit by accounting for breeding values, weightings for traits of economic importance and ranking sires and cows within breeds. Various dairy profit indexes currently exist and are briefly described in the following paragraphs. The UK Profitable Lifetime Index (£PLI) is a within-breed genetic ranking index that accounts for production (34.4%), survival (15.1%), efficiency (11.8%), calving ability (1.6%), leg health (8.1%), udder health (13.7%) and fertility (15.3%; AHDB, 2020). The £PLI places emphasis on promoting milk yield and maintaining milk quality for additional profit for UK dairy farmers with all year-round calving herds, and has two sub-indexes: the Spring Calving Index (£SCI) and the Autumn Calving Index (£ACI). Both sub-indexes are across-breed genetic ranking indexes designed for spring block calving herds and autumn block calving herds, respectively.

Canada’s Lifetime Profit Index (LPI) accounts for 50% genetic plan on production, 30% durability and 20% health and fertility (CDN, 2021). The LPI formula for each breed is applied to bulls and cows in Canada that participate in national genetic evaluations for production and type trait and are used to compute MACE for sires in most global dairy sectors (CDN, 2009; Interbull, 2013). The Australian Profit Index (API), a prototype of the Balanced Performance Index (BPI) is a profit-based production index that accounts for nine traits such as milk, fat and protein yields, live weight, somatic cell count, fertility, survival, temperament and milking speed (Valentine et al., 2000). The updated API currently includes an economically optimal solution for farmer trait preferences with increased emphasis on fertility and fitness (Pryce et al., 2004).

The Dutch milk product index also known as the total merit index of the Netherlands and Flanders (NVI) puts a lot of weight emphasis on production (40%), longevity and health (35%) and type (25%).

The American Net Merit Index (NM$) also known as Lifetime Net Merit (NM$) ranks dairy animals based on their combined genetic merit for economically important traits. The NM$ contains three major trait categories; production (45%), health (40%) and type (15%) (Table 3). These major traits are updated periodically by the Council on Dairy Cattle Breeding (CDCB, 2021) to include genetic evaluations for single and composite traits (Liang et al., 2017; VanRaden, 2017). As an example, three other traits were incorporated into the updated 2021s NM$ and this includes; feed saved, heifer livability, and early first calving (VanRaden et al., 2021). It is expected that selection for new traits and future selection of economically important traits will improve health, growth of calves, production, fertility, feed efficiency of cows, and reflect prices anticipated in the future for American dairying (Cole et al., 2021).

**TABLE 3:** International indices with proportion of relevant traits (%) to the proposed dairy profit index for Rwanda.

Index type	Production	Fertility	Body weight/Growth	Survival/longevity/stayability	Efficiency	Calving ability	Leg health	Udder health	Conformation			Milk fat	Milk protein	Milk volume	BCS	SCS
									Udder	Feet and legs	Claw health					
ABEA index			10	11								24	17	13	7	6
Canadian LPI$	51	7.5		34			7.5									
New Zealand’s BW			11	9								24	17	13	7	6
£PLI	34.4	15.3		15.1	11.8	1.6	8.1	13.7								
Dutch milk product index	40			35				25								
INET	29	16		12	8			12	5	9	7					
NM$	45						40		15							
TPI	46	28							13	13						
INEL of France	50	12.5		12.5					12.5							12.5
Jersey SAINET	55								10	35						
Holstein BVI	52							3	45							
Scandinavian NTM	40	22.5							15							
							22.5									
Tanzania index	50														50	

£PLI, UK profitable lifetime index; £ACI, autumn calving index; £SCI, spring calving index; Canadian LPI$, Canadian lifetime profit index; INET, net profit index for milk production; NM$, Net Merit Index; TPI, Total Production Index; Holstein BVI, breeding value index; BW, New Zealand’s breeding worth; Scandinavian NTM, Nordic total merit.

The Dairy Wellness Profit Index (DWP$) was launched by Zoetis^®^ as a unique and comprehensive animal ranking selection index that lays emphasis on the value of critical wellness and health traits. The DWP$ offers very similar selection emphasis to NM$ traditional traits but with additional selection emphasis on wellness traits to make more comprehensive and profitable genetic selection decisions. The DWP$ is the only index included in CLARIFIDE plus^®^ for ranking and genomic testing of animals against six common health challenges such as: mastitis, lameness, metritis, retained placenta, ketosis and displaced abomasum (Zoetis^®^, 2018) to enhance herd health, marketable milk and overall herd profitability.

The Total Production Index (TPI) of the Holstein Association USA (HAUSA) lays more emphasis on Production; 46%, 28% Health and 26% Conformation (Table 3). The TPI is also updated periodically to reflect current research trends and genetic evaluations for new traits that have been made available to the dairy industry. For instance the current TPI HAUSA includes a modification to the existing Feed Efficiency (FE$) to include the new Feed Saved trait (HAUSA, 2022). This ensures greater feed efficiency through improved production, feed saved from cows with a lower body weight, better feed conversion and less maintenance costs.

The Index Economique Laitier (INEL) index of France also referred to as the economic dairy yield index, puts more emphasis on production (50%) than fertility, somatic cell count, longevity and morphology/conformation (each at 12.5%). The INEL ensures that dairy quality, productivity and profitability are increased by hinging on minimising costs of veterinary bills, breeding and reproduction costs.

The two main dairy indexes used for selection of dairy traits of economic importance in South Africa are the; Jersey SAINET and Holstein Breeding Value Index (BVI) (Banga, 2009, PhD thesis). The Jersey SAINET is a South African index (Taurus Jersey, 2007) that favours production and linear-type traits. The index is further divided to three sub-indexes; production index (55%), functional udder index (10%) and functional type index (35%). The South African Holstein Breeding value index (BVI) is a production-type index, favouring high protein and butterfat producing cows, with large framed and extremely angular bodies, and, tightly attached udders. The BVI considers 52% production, 45% functional type trait and 3% on udder health (Taurus Holstein, 2007) (Table 3). However, the Jersey and Holstein indexes are not widely adopted within the country’s dairy sector due to a lack of consensus on the appropriate dairy traits of economic importance for inclusion in dairy breeding goals. The authors of this report recognise that in countries where there may be multiple management systems, it is often difficult to create a single index which supports all systems. In large part it is this recognition that enables us to focus this section of the review on the development of a simple dairy profit index that primarily focuses on the development of dairy in a smallholder system environment. Banga (2009) proposed that a single breeding objective on the basis of multiple-trait selection for South Africa’s major dairy breeds would be useful across the different production and economic payment systems. However, considerable progress is required to enhance this breeding objective as well as facilitate its wide adoption within-country and other countries in Africa.

The Nordic Total Merit Index (NTM), a Scandinavian index, is one of the most progressive breeding value systems in the global dairy industry that combines 90 different sub-indexes into 15 different genetic traits that are heritable through mating bulls with cows. The Jersey breed NTM lays emphasis on health and reproduction (45%), production efficiency (40%) and conformation and workability (15%) (Viking Genetics, 2021). The aim is to develop the cattle’s genetic and financial potential to achieve higher profitability and functionality of the herd by breeding new generations of cows with higher capability e.g. for milk production and resistance to diseases. The index also focuses heavily on management and health traits as it draws on the extensive dataset for these traits collected on Scandinavian dairy farms by law.

The New Zealand Index, also known as Breeding Worth is an index that accounts for 24% milk fat, 17% protein, 13% milk volume, 11% live weight, 13% fertility, 6% somatic cell score, 9% residual survival and 7% body condition score (Dairy New Zealand, 2021). The index accounts for milk production, feeding efficiency and grazing ability, robustness, minimal heifer replacement, survival of dairy cows and sires for future genetic breeding strategies for farm profit. Therefore, the Breeding Worth Index’s high focus on fertility, milking ability and production per Kg live weight are of great relevance to the implementation of a proposed dairy (suitability) profit index for Africa. The coordinated and comprehensive data recording and genetic evaluation system in New Zealand is one of the critical factors that has increased the economic efficiency and viability of genetic improvement in the dairy industry. Therefore, the New Zealand Dairy Profit Index could be relevant and applicable to the development of an index mechanism for countries in Africa. In addition, New Zealand has genetically and genomically sampled many dairy cattle strains of the black and white breeds (i.e. Holstein and Friesian breeds); the red and white Scandinavian breeds to many of the Jersey populations originating from North America and Europe, as well as the Sahiwal breed native to Asia. All these breeds are more frequently now found as relatively pure or crossbred genotypes in Africa and the tropics.

A characteristic New Zealand-type dairy cattle, whether pure or crossbred, is small or moderate in size, matures earlier and has inherently higher fertility characteristics than dairy cattle in other populations. Such cows are pasture-based and have been developed over many generations to suit a very specific management system which may differ from dairy cattle found in other systems (Blackwell et al., 2010; Gardner, 2017). Crossbreeding is predominantly used for dairy production in both New Zealand and Africa whereby the genetic evaluation system analyses all breeds together so that the breeding values and profitability of crossbreds and purebreds can be referenced and compared directly across all breed genotypes. The New Zealand’s Breeding worth and UK’s Spring Calving Index have similar characteristic components as both are described as an across-breed index with exclusive reliance on pasture or grass feeding in conjunction with reducing maintenance costs and improving fertility, production, feed efficiency, conformation, survival and longevity. In addition, the generally less-intensive nature of dairy farm management practices in New Zealand has resulted in dairy cows that could be more suitable to Africa’s milk production systems. Dairy cattle in New Zealand and many smallholder dairy systems in Africa get little cereal grains or other supplementary feed stuff. However, in South Africa where commercial dairying is developed, often utilise high cereal grain feed (TMR – Total Mixed Ration). Dairy cattle in New Zealand being more feed efficient have to produce as much milk as possible primarily from grass based on seasonal growth patterns, and then optimise their productivity, whilst their inherent enhanced fertility advantage better enables them to secure a pregnancy to calve again within the tight re-calving pattern required (Lopez-Villalobos and Garrick, 2006).

### Development of dairy profit indexes applicable to African production systems

A proposed (all-breed type) index for Africa that draws elements from the New Zealand type index, East Africa index and the UK’s spring calving index to include increased weightings for fertility, calving ease, reduced condition loss and replacement costs, and disease resistance to mastitis would be an initial step, the index could then be modified as more information is recorded and included. An index in Africa could enhance the financial value returns of animals as it provides the basis by which animals can be ranked enabling farmers to choose the appropriate cow that fit the diverse management systems in Africa.

In selected African countries, several researchers have previously performed genetic evaluation (Dube et al., 2009; Missanjo et al., 2013; Madilindi et al., 2019; Opoola et al., 2020) and most recently, genomic evaluations on the Jersey breed (Chagunda et al., 2018). Preliminary methods and statistical procedures such as least squares mean and generalised or mixed linear models have been used in data description and curation for onward data analyses (Cunningham and Syrstad, 1987; Nouala et al., 2003; Opoola et al., 2019). Parameter estimations such as variance components, heritabilities and genetic correlations for MY, AFC, calving interval, feed efficiency, adaptability and disease resilience have been determined using residual maximum likelihood approach in both biological and genetic software programmes (Nouala et al., 2003; Dube et al., 2009; Missanjo et al., 2013; Ojango et al., 2019). The estimations of these parameters for the aforementioned traits from performance data records provides opportunities to monitor genetic progress over a time period as well as optimise the implementation of sustainable breeding programmes using information available for the breed (Asimwe and Kifaro, 2007; Mostert et al., 2010; Makina, 2015; Opoola et al., 2020).

A proposed dairy profit index (DPI) that include traits of economic importance that also addresses current challenges faced by the African dairy sector will help maximise dairy productivity and improve efficiency of breeding plans for increased profits to dairy farmers. The East Africa dairy profit index developed by the Animal Breeding East Africa Ltd (ABEA, 2022) that draws elements from the New Zealand’s Breeding Worth index is a good starting point for developing individual country indexes. The index developed for each country may be different in terms of monetary currency, input and output costs. However, the criteria for selection of measurable traits of interest (e.g. production, fertility, growth, survival and disease influence, *etc.*) would be similar across the countries in Africa even though sire breeding values could be different due to influence of GxE.

A proposal for a Rwanda DPI would include economic weightings for measurable traits for milk yield, fertility, growth and survival, herd health and disease resistance, longevity and conformation whereby bulls and cows with known breeding values and genomic breeding values are selected on the current breeding plan.

The traits measured should include:

1. Production; daily milk yield, total days in milk, lifetime milk yield and annual milk yield.
2. Fertility; for both cow and bull traits such as AFC, calving ease, calving interval, non-return rate, milk yield around insemination, days from calving to first insemination, number of inseminations per conception and days open.
3. Survival; in terms of longevity, cow/herd life stayability in the herd and reduced culling rate.
4. Health; such as number of health interventions, incidence of mastitis, lameness and vector-borne diseases.
5. Growth and conformation; Liveweight and body condition score.

The existence of other global dairy indexes and decision-support tools based on priority traits guides us towards building the necessary information for developing a selection index tool for Africa (or Rwanda as an exemplar). Such index development for Rwanda could optimise milk yield, fertility and body weight by ranking of suitable dairy breeds for Africa (Table 3). Table 3 shows some of the dairy profit indexes of relevance to the proposed index for Rwanda. Most of the traits have proportions assigned with respect to performance, fertility, and conformation and including health traits. The proposed decision-support tool (dairy profit Index) will be derived using both performance (phenotype) data records as well as genotype information for milk yield, fertility and body size already accounted by growth. In addition, ranking procedures that include economic weights for input costs, management and the EBV and GEBVs for the components that make up milk yield, fertility and body size in relation to growth could provide initial information for the proposed DPI for Rwanda. The derived GEBVs will guide in selecting breeding candidates and ranking bulls with favourable traits. The proposed decision-support at its first inception is expected to be an open-ended dairy profit index whereby more traits of economic importance will be included as the performance recording systems matures and data become available.

## Conclusions and perspectives

This review highlights impacts, performances and activities of the Jersey breed in African countries. Although there is a paucity of detailed historical information about the Jersey breed in some African countries, the performance of the Jersey breed where it has been found or currently resides clearly shows the potential of exploring the breed’s influence in Africa’s dairy production systems. Therefore, whilst building a reference population for genomic selection of all exotic breeds currently used for dairy production in Africa could help drive productivity and profit for smallholder farmers, a reference population that links small or moderately sized cows like the Jersey breed, to traits of economic importance, could help inform future breeding strategies for smallholder farmers in developing countries especially.

To our knowledge, this paper is the first review of the Jersey cow in Africa and summarises available information on its performance, and other characteristics to support options for sustainable dairy development strategies. However, the data gap remains a challenge in many countries. There is a growing interest for breed assessment and recording of exotic breeds for future dairy improvement. Such systems should not be dependent on individual grant-funded or research projects being executed but need to involve both government and private partnerships and must provide decision support systems to farmers to improve livestock management in addition to improved genetics. It is encouraging, however, to note that livestock data collection and technical support has been a key driver for the past 5 years in the development of animal agriculture in Africa (Marshall et al., 2019; Ojango et al., 2019; Okeyo, 2021).

In addition to the more focused data collection and genomic sampling that has commenced in Rwanda (led by RJAHS, CTLGH, RAB, with others), other dairy programmes both in Rwanda (e.g. the Rwanda Dairy Development Project) and elsewhere (e.g. the African Dairy Genetic Gains (ADGG) platform) have established innovative systems with long-term objectives including genomic sampling and data collection on dairy performance. More data will further support long-term genetic improvement, based on established breeding programmes to maximise the breed’s genetic potential. This will also offer the opportunity to establish a set of markers for genomic selection and breeding values that are associated with economically and environmentally important traits for specific ecologies and production systems.

The global indexes used in advanced economies are complex and not directly applicable in Africa. They require considerable and sustainable data collection, which is unlikely to happen in smallholder East African production systems at the present time. However, with the African dairy sector progressing towards a more sustainable system of production, through adequate performance data recording to monitoring genetic progress, there would be a possibility of developing a dairy profit index, tailored to Africa’s smallholder farmers to optimise dairy productivity.

In addition, an index that best defines suitability and adaptability as seen in other indexes used in advanced economies could help the dairy sector in Africa because of the following reasons:

1. To contribute to the identification and selection of suitable bulls for use in the African smallholder dairy systems.
2. To improve our understanding of the GxE effects in our targeted production systems.
3. Additional benefits, could be the ability to combine both phenotypic and genotypic data, generate estimated breeding values, to support ranking of genetics suitable for the production systems, and support exchange/trade of genetics among African countries.
4. It will also enhance the availability for cattle of specific breeds within countries, to help guide future breeding policies.

Similarly, and in pursuance of these aims, the RJAHS is collaborating with RAB to ensure smallholder farmers in Rwanda have access to what are anecdotally considered to be the more appropriate Jersey genotypes for the country’s smallholder systems. In addition, livestock data will be tracked and traced from farm to an online database system, where uniform performance data recording will promote and monitor genetic progress. This is being further supported by the genomic profiling of Rwanda’s current dairy cattle genetics.

We anticipate that these efforts will contribute to dairy cattle that are both more profitable and more intrinsically suited to the environment in which they are being asked to perform. For Rwanda these socio-environmental factors include a cow that often needs to be managed and handled by the female in the household; that will need to survive climatic, disease and other health challenges; produce a nutrient rich foodstuff (milk or dairy products) from limited forage-based feed resources, and maintain sufficient body condition to rebreed and carry a calf.

A well-structured approach to future dairy cattle breeding policy that is developed around economically important dairy traits in the profit index, where animals with improved appropriate genetic merit are recognised, and financial returns are optimised is the recommended route to improving dairy farming sustainability for smallholder farmers. This is a target that we should all strive for, while recognising that the Jersey breed is likely to hold the key to solving a number of these challenges.

## Data Availability

The original contributions presented in the study are included in the article/supplementary materials, further inquiries can be directed to the corresponding authors

## Footnotes

1 Standard Error.

2 Not significant.

3 Not significant.
